# Utilization of ACP CPT codes among high-need Medicare beneficiaries in 2017: A brief report

**DOI:** 10.1371/journal.pone.0228553

**Published:** 2020-02-05

**Authors:** Amanda J. Reich, Ginger Jin, Avni Gupta, Dae Kim, Stuart Lipstiz, Holly G. Prigerson, Jennifer Tjia, Keren Ladin, Scott D. Halpern, Zara Cooper, Joel S. Weissman

**Affiliations:** 1 Center for Surgery and Public Health, Brigham and Women’s Hospital, Boston, MA, United States of America; 2 Department of Health Policy, College of Global Public Health, New York University, New York, NY, United States of America; 3 Marcus Institute for Aging Research, Hebrew SeniorLife, Boston, MA, United States of America; 4 Cornell Center for Research on End-of-Life Care, Weill Cornell Medicine, New York, NY, United States of America; 5 Population and Quantitative Health Sciences, University of Massachusetts Medical School, Worcester, MA, United States of America; 6 Research on Ethics, Aging, and Community Health (REACH), Tufts University, Boston, MA, United States of America; 7 Palliative and Advanced Illness Research (PAIR) Center, University of Pennsylvania, Philadelphia, PA, United States of America; 8 The Division of Pulmonary, Allergy, and Critical Care Medicine, Department of Medicine, University of Pennsylvania, Philadelphia, PA, United States of America; University Complutense of Madrid, SPAIN

## Abstract

**Importance:**

Medicare beneficiaries with high medical needs can benefit from Advance Care Planning (ACP). Medicare reimburses clinical providers for ACP discussions, but it is unknown whether high-need beneficiaries are receiving this service.

**Objective:**

To compare rates of billed ACP discussions among a cohort of high-need Medicare beneficiaries with the non-high-needs Medicare population.

**Design:**

Retrospective analysis of Medicare Fee-for-Service (FFS) claims in 2017 comparing high-need beneficiaries (seriously ill, frail, ESRD, and disabled) with non-high need beneficiaries.

**Setting:**

Nationally representative FFS Medicare 20% sample

**Participants:**

Medicare beneficiaries were assigned to one of the following classifications: seriously ill (65+), frail (65+), seriously ill & frail (65+); non-high need (65+); end stage renal disease (ESRD) or disabled (<65). All participants had data available for years 2016–2017.

**Exposure:**

Receipt of a billed ACP discussion, CPT codes 99497 or 99498.

**Main outcome and measure:**

Rates of billed ACP visits were compared between high-need patients and non-high-need patients. Rates were adjusted for the 65+ population for sex, age, race/ethnicity, Charlson comorbidity index, Medicare/Medicaid dual eligibility status, and Hospital Referral Region.

**Results:**

Among the 65+ groups, those most likely to have a billed ACP discussion included seriously ill & frail (5.2%), seriously ill (4.2%), and frail (3.3%). Rates remained consistent after adjusting (4.5%, 4.0%, 3.1%, respectively). Each subgroup differed significantly (p < .05) from non-high need beneficiaries (2.3%) in both unadjusted and adjusted analyses. Among the <65 high need groups, the rates were 2.7% for ESRD and 1.3% for the disabled (the latter p < .05 compared with non-high needs).

**Conclusions and relevance:**

While rates of billed ACP discussions varied among patient groups with high medical needs, overall they were relatively low, even among a cohort of patients for whom ACP may be especially relevant.

## Introduction

Advance Care Planning (ACP) aims to align medical treatment to patients’ values, goals and preferences for care during serious and chronic illness.[1],[2] Medicare is the United States federal health insurance program designed to cover individuals over age 65, and under age 65 who have a qualifying disability or end-stage renal disease (ESRD). Effective January 1, 2016, Medicare (CMS) reimburses clinical providers for ACP. Discussions eligible for reimbursement by Medicare include explanation and discussion of advance directives such as standard forms, which may result in documentation of patient preferences for end-of-life (EOL) treatment. ACP includes a discussion between a qualified healthcare provider and a patient, and is an important component of care management. Medicare does not require any specific beneficiary diagnosis to reimburse for ACP discussions, and any beneficiary is entitled to the service. A review of best practices of serious illness care communication defined ACP “high value care advice” as conversations that should begin as early as possible in the course of serious or life-limiting illnesses. Care management is especially complex for “high-need” patients, who face a combination of high healthcare costs, utilization intensity, and functional limitations. A recent analysis of interventions and policies related to ACP recommended a focus on the seriously ill to improve value of care towards the end of life.[3] Given that roll-out of ACP billing procedures may require investment of time and money by health systems, timing ACP strategically via prognostic stratification of patients can help to identify groups at higher risk for facing end-of-life decisions, [4] and may improve quality of life, family outcomes, and reduce costs.[5],[6] Prior research on uptake of the ACP billing codes [7],[8] has not addressed these patients. This analysis therefore examined rates of billed ACP discussions among a national cohort of high-need Medicare patients compared with Medicare beneficiaries not identified as high need. We hypothesized that high needs patients of all types would have higher rates of billed ACP discussions than other patients.

## Methods

### Population

The Partners Human Research Committee reviewed this research and determined it is "not human research", approval protocol number 2017P000371/PHS. We used the 20% sample of Fee-For-Service (FFS) Medicare beneficiaries from 2016–2017. Beneficiary characteristics were obtained from the Medicare beneficiary summary file. We used claims in 2016 to identify high-need patients and claims in 2017 for receipt of a billed ACP visit. Beneficiaries alive in 2017 were categorized into six (6) mutually exclusive groups, stratified by age: 1) seriously ill only age 65+ (SI) 2) frail only 65+ (F); 3) beneficiaries who are both frail and seriously ill 65+ (F&SI); 4) non-high-need 65+; 5) disabled <65; 6) end-stage renal disease (ESRD) <65. The high needs groups in this analysis are closely aligned with those described by the National Academy of Medicine (NAM).[9] We excluded beneficiaries whose first billed ACP discussion occurred in 2016 as the visit may have coincided with the qualifying eligibility criteria.

There is no consensus definition of serious illness.[10] Therefore, we identified criteria for serious illness using ICD-10 codes to capture patients with a median survival of roughly two years or less and/or substantial suffering. Focusing on high risk of mortality provides an opportunity to target patients with persistently high healthcare utilization.[11] We included the primary and secondary diagnoses of chronic obstructive pulmonary disease (COPD) and other lung diseases, heart failure, renal failure, cancer, dementia, and neurodegenerative diseases in the cohort (See randomized trials NCT02100566, NCT02487810, NCT02505035, NCT02017548).

To identify frailty, we adapted the ICD-10 codes from the Claims-based Frailty Index,[12] including abnormality of gait, abnormal loss of weight, adult failure to thrive, cachexia, debility, difficulty in walking, history of fall, malaise and fatigue, muscular wasting, muscle weakness, pressure ulcer, and senility without mention of psychosis. In addition, we used HCPCs codes to capture patients using durable medical equipment. Any beneficiaries with at least two or more of these ICD-10 codes in 2016 were considered frail.

We used the Disability Insurance Benefits (DIB) and End-Stage Renal Disease (ESRD) Medicare entitlement to select beneficiaries with a disability or ESRD. Beneficiaries who qualify under age 65 due to a permanent disability have relatively high rates of chronic conditions, functional limitations, and cognitive impairments[13]. The ESRD category included a small number of beneficiaries with DIB. We identified these beneficiaries by their “current” status, so all were under age 65.

### Outcomes

The primary outcome was the receipt of one or more billed ACP discussions, defined as having at least one visit that included either primary Current Procedural Terminology (CPT) code 99497 or 99498 between January 1 and December 31, 2017. CPT codes are used by Medicare to determine the amount of reimbursement that a practitioner will receive for each service. Code 99497 reimburses for the first 30 minutes of ACP discussions with patients, family members, and/or surrogates, and the add-on secondary code 99498 reimburses for extended time beyond 30 minutes.

### Statistics

We conducted the analyses to compare utilization of the ACP billing code for the high-need groups with all the non-high need population. We first calculated the crude billed ACP discussion rates without adjustment for each group. In the multivariate analysis, we restricted it to those who were at least age 65 since the comparison group of non-high-needs population was 65+, we further computed the adjusted rates for the seriously ill, frail, seriously ill & frail to all non-high-need beneficiaries from multivariate logistic regression models approximately by the SAS LESMEANS statement, after accounting for all potential confounders. Covariates considered for the 65+ population included sex, age (65–74, 75–84, 85+), race/ethnicity (Non-Hispanic White, Non-Hispanic Black, Hispanic, Asian, other, unknown), Charlson comorbidity index (CCI) calculated using a 12-month look-back period (0, 1, 2, 3, 4, 5 or more), Medicare/Medicaid dual eligibility status, and Hospital Referral Region (HRR) level of overall spending. We categorized HRRs according to total spending from 2012–2016[14], adjusted for race, sex and age, as ‘high’ (>75^th^), medium (25 to 75^th^), and low (<25^th^) percentiles.

All analyses were conducted using SAS software (version 9.4, SAS, Cary, NC). We adopted the Pearson’s chi-square test for categorical variables and Wilcoxon’s test for the continuous variables, to compare the baseline characteristics. Two-sided P-values <0.05 were considered statistically significant.

## Results

### Sample

The cohort included 5,766,056 beneficiaries, including non-high-need beneficiaries (4,085,666), seriously ill (N = 429,841), frail (N = 158,939), seriously ill & frail (N = 172,472), ESRD (N = 15,596) and disability (N = 903,542). We excluded 30,545 whose first billed ACP discussion occurred in 2016. Compared to the non-high needs group, higher proportions of seriously ill, frail, and seriously ill & frail beneficiaries were female and white, while higher proportions of beneficiaries with ESRD or disability were male, Black, and have dual-eligibility status (Table 1, all p < .05). The CCI was lowest in the disability group. Finally, compared to non-high-needs beneficiaries, larger proportions of all high-need subgroups except those with a disability were more likely to reside in higher spending HRRs.

**Table 1:** Characteristics of included beneficiaries (2017).

	All (N = 5,766,056)		Non-high-needs (N = 4,085,666)		age 65+						under age 65			
					SI (N = 429,841)		Frail (N = 158,939)		SI & Frail (N = 172,472)		ESRD (N = 15,596)		Disability (N = 903,542)	
	n	%	n	%	n	%	n	%	n	%	n	%	n	%
ACP*	139201	2.4	94,838	2.3	18,204	4.2	5,259	3.3	8,957	5.2	415	2.7	11,528	1.3
**Sex**														
Male	2,597,169	45	1,799,297	44	200,986	46.8	52,152	32.8	65,645	38.1	9,014	57.8	470,075	52
Female	3,168,887	55	2,286,369	55.9	228,855	53.2	106,787	67.2	106,827	61.9	6,582	42.2	433,467	48
**Age**														
<65	873,999	15.2	#	#	#	#	#	#	#	#	15,038	96.42	858,961	95
65–74	2,373,812	41.2	2,112,309	51.7	133,434	31	44,821	28.2	38,109	22.1	#	#	#	#
75–84	1,681,963	29.2	1,395,767	34.2	167,002	38.8	57,510	36.2	61,684	35.8	#	#	#	#
> = 85	836,282	14.5	577,590	14.1	129,405	30.1	56,608	35.6	72,679	42.1	#	#	#	#
**Race**														
White, non-Hispanic	4,824,119	83.7	3,510,427	85.9	363,297	84.5	141,214	88.8	149,117	86.5	6,251	40.1	653,813	72.4
Black, non-Hispanic	526,229	9.1	282,209	6.9	41,674	9.7	10,863	6.8	15,979	9.3	6,237	40	169,267	18.7
Asian/North Amer Native	135,853	2.4	96,862	2.4	9,407	2.2	2,578	1.6	2,843	1.6	987	6.3	23,176	2.6
Hispanic/Latino	110,528	1.9	59,537	1.5	7,158	1.7	1,711	1.1	2,313	1.3	1,535	9.8	38,274	4.2
Other	91,129	1.6	71,881	1.8	5,753	1.3	1,578	0.9	1,544	0.9	279	1.8	100,094	1.1
Unknown	78,210	1.4	64,750	1.6	2,552	0.6	995	0.6	676	0.4	307	2	8,918	1
**Dual Eligibility Status**														
No	4607959	79.9	3,662,754	89.6	335,930	78.1	119,540	75.2	112,655	65.3	5,343	34.3	371,065	41.1
Yes	1159374	20.1	422,912	10.3	93,911	21.8	39,399	24.8	59,817	34.7	10,253	65.8	532,477	58.9
**Charlson Index**														
0	3,366,389	58.4	2,718,409	66.5	74,073	17.2	42,631	26.8	7,378	4.3	688	4.4	523,210	57.9
1	1,178,234	20.4	834,206	20.4	77,281	18	48,401	30.4	22,013	12.8	4,033	25.9	192,300	21.3
2	582,517	10.1	335,965	8.2	84,614	19.7	34,389	21.6	32,338	18.7	2,890	18.5	92,321	10.2
3	307,986	5.3	131,667	3.2	73,773	17.2	19,254	12.1	33,524	19.4	2,645	17	47,123	5.2
4	164,811	2.9	46,198	1.1	54,312	12.6	9,162	5.8	28,821	16.7	2,082	13.3	24,236	2.7
5 or above	166,119	2.9	19,221	0.5	65,788	15.3	5,102	3.2	48,398	28.1	3,258	20.9	24,352	2.7
**HRR Overall Spending**														
High	1,499,282	26.1	1,042,454	25.6	133,433	31.1	37,737	23.8	51,948	30.1	5,026	32.6	228674	25.4
Medium	3,161,670	55.1	2,234,112	54.9	230,690	53.9	91,761	57.8	96,939	56.2	8,216	53.4	499952	55.5
Low	1,080,025	18.8	788,883	19.4	64,187	15	29,313	18.5	23,464	13.6	2,153	14	172025	19.1

*ACP = beneficiaries with at least one billed ACP visit in 2017 (in high-need and 20% FFS cohorts# = N/A

HRR refers to Dartmouth Atlas Health Referral Regions, SI = Seriously Ill, ESRD = end stage renal disease. Totals will vary due to missing values

### ACP visits

**Fig 1:**
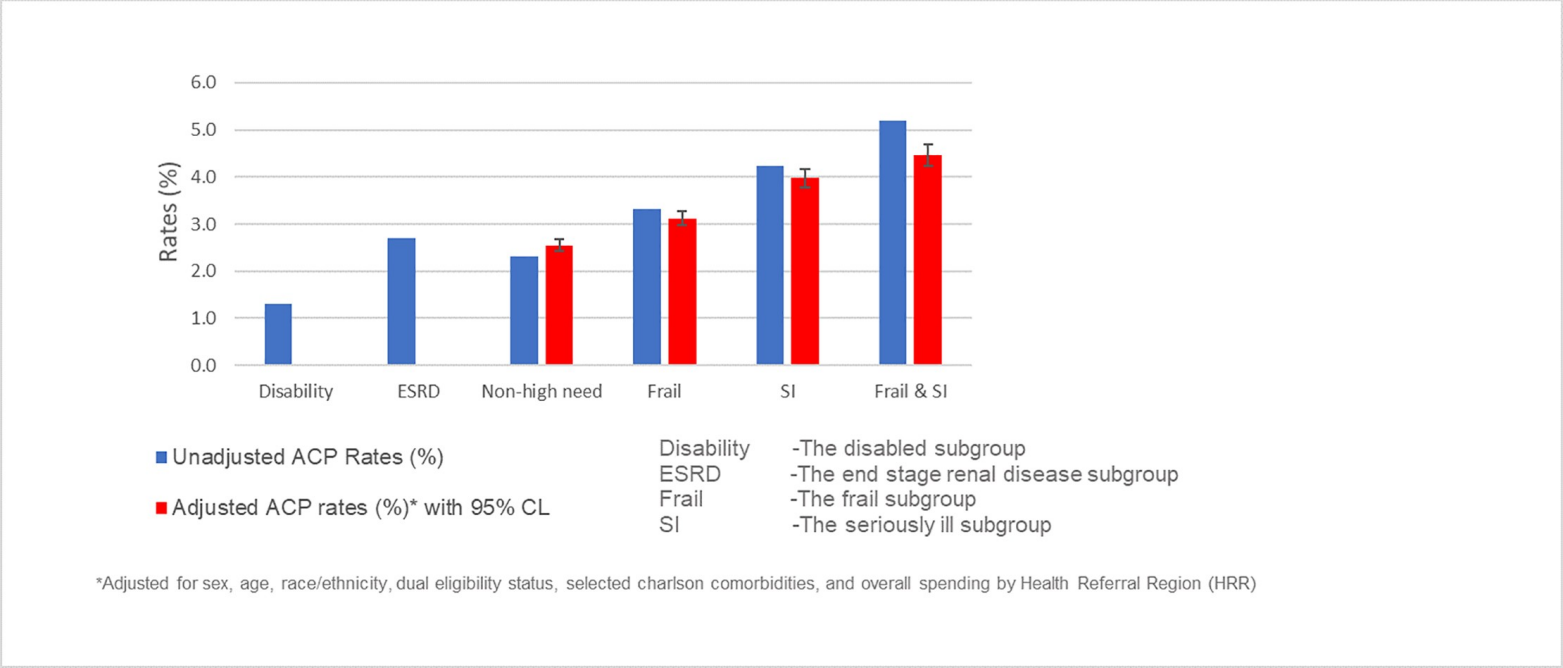
Rates of ACP visits among high-need and non-high need Medicare beneficiaries (2017.

Overall rates of billed ACP discussions were low, 2.4%, in 2017 2.3% among non-high-needs beneficiaries). ACP billing varied among the 65+ groups, 2.3% of non-high-need beneficiaries compared to 5.2% of seriously ill & frail, 4.2% of seriously ill, and3.3% of frail beneficiaries. (See Fig 1). Rates remained consistent after adjusting (4.5%, 4.0%, 3.1%, respectively). Each subgroup differed significantly (p < .05) from non-high needs beneficiaries in both unadjusted and adjusted analyses. Among the <65 high needs groups, the rates were 2.7% for ESRD and 1.3% for the disabled (the latter p < .05 compared with non-high needs).

## Discussion

We found overall low rates of billed ACP discussions among high needs beneficiaries. Still, there are important distinctions. Specifically, beneficiaries with a diagnosis of disability or ESRD were less likely than other high needs groups, and among disabled beneficiaries, even the average Medicare patient, to receive a billed ACP discussion. By contrast, beneficiaries designated as seriously ill or frail were more likely than other patients to have a billed ACP discussion.

Beneficiaries who met the criteria for being both frail and seriously ill were potentially the sickest group, and in fact were the most likely to receive a billed ACP discussion. For the combined seriously ill & frail group, the rate was 5.2% unadjusted, 4.4% adjusted. While these rates were among the highest in the cohort, they are still relatively low.

ESRD beneficiaries may be an important population group to include in targeted ACP interventions. A study comparing decedents with ESRD to cancer patients found lower rates of ACP conversations and higher treatment intensity at the end of life among ESRD patients, despite similarly high rates of symptoms[15]. ESRD beneficiaries also have been shown to receive palliative care consultation services less often than other seriously ill patients who have comparable symptom burdens.[16]

Existing research on ACP among disabled Medicare beneficiaries is limited. ACP discussions may be especially complex due to a history of discrimination of people with disabilities and disparities in their care.[17],[18]

The content of ACP discussions varies across the life and health trajectory, and differences among these high-need beneficiaries may be driven in part by the diversity of age and health status within the cohort. In designing interventions to better align care with the goals of high-need patients during billed ACP discussions, health systems must recognize that different high-need patient segments require different services and workforce competencies.[9] Thus, while focusing efforts at increasing ACP among all the identified subgroups is an important strategy for directing limited resources, the discussions will vary between patients who are seriously ill and may focus on end-of-life issues, and a younger, relatively healthy beneficiary with a disability who may need to designate a proxy but for whom specific end-of-life decision making is less immediately relevant.

## Limitations

This study has several limitations. This study relies on claims data, and while initial uptake of the billing code is low, the absence of an ACP billing code does not mean that an ACP discussion has not occurred. Further, administrative claims data do not provide data on the quality of the discussion, and we cannot assess whether care provided was congruent with patient’s wishes via claims data alone. Finally, further research is needed to determine whether ACP discussions impact the course of care.

## Conclusions

While rates of billed ACP discussions vary among high-need Medicare beneficiaries based on categories of high need, overall use of the code remains low. Further examination of ACP billing codes may be helpful in targeting healthcare resources to improve ACP among high need patients.

ACP discussions should reflect an individual’s health and personal preferences. When implementing system-wide strategies to build capacity and increase ACP discussions, providers should be equipped with appropriate training in communication informed by best practices guidelines.[5] Health systems can improve care for high-need patients by prioritizing these subgroups for ACP discussions, but should ensure that the distinct needs between patients are accommodated.
